# Bone marrow fibrosis in myelodysplastic syndromes: a prospective evaluation including mutational analysis

**DOI:** 10.18632/oncotarget.9026

**Published:** 2016-04-26

**Authors:** Fernando Ramos, Cristina Robledo, Francisco Miguel Izquierdo-García, Dimas Suárez-Vilela, Rocío Benito, Marta Fuertes, Andrés Insunza, Eva Barragán, Mónica del Rey, José María García-Ruiz de Morales, Mar Tormo, Eduardo Salido, Lurdes Zamora, Carmen Pedro, Javier Sánchez-del-Real, María Díez-Campelo, Consuelo del Cañizo, Guillermo F. Sanz, Jesús María Hernández-Rivas, Spanish Group for Myelodysplastic Syndromes (GESMD)

**Affiliations:** ^1^ Department of Hematology, Hospital Universitario de León, León, Spain; ^2^ Instituto de Biomedicina (IBIOMED), Universidad de León, León, Spain; ^3^ Unidad de Diagnóstico Molecular y Celular del Cáncer, IBSAL, IBMCC-Centro de Investigación del Cáncer (USAL-CSIC), Salamanca, Spain; ^4^ Department of Pathology, Hospital Universitario de León, León, Spain; ^5^ Department of Pathology, Hospital Valle del Nalón, Langreo-Asturias, Spain; ^6^ Department of Hematology, Hospital Universitario U. Marqués de Valdecilla, Santander, Spain; ^7^ Department of Molecular Pathology, Hospital Universitari i Politècnic La Fe, Valencia, Spain; ^8^ Department of Immunology, Hospital Universitario de León, León, Spain; ^9^ Department of Hematology-Oncology, Hospital Clínico Universitario, Valencia, Spain; ^10^ Department of Hematology, Hospital Universitario Virgen de la Arrixaca, Murcia, Spain; ^11^ Unit of Molecular Genetics, ICO-Hospital Germans Trias i Pujol, Institut de Recerca contra la Leucèmia Josep Carreras, Badalona, Spain; ^12^ Department of Hematology, Hospital del Mar, Barcelona, Spain; ^13^ Department of Hematology, Hospital Universitario de Salamanca, Spain; ^14^ Department of Hematology, Hospital Universitari i Politècnic La Fe, Valencia, Spain

**Keywords:** myelodysplastic syndromes, bone marrow fibrosis, next-generation sequencing, pathogenesis, prognosis

## Abstract

The biological and molecular events that underlie bone marrow fibrosis in patients with myelodysplastic syndromes are poorly understood, and its prognostic role in the era of the Revised International Prognostic Scoring System (IPSS-R) is not yet fully determined. We have evaluated the clinical and biological events that underlie bone marrow fibrotic changes, as well as its prognostic role, in a well-characterized prospective patient cohort (*n*=77) of primary MDS patients. The degree of marrow fibrosis was linked to parameters of erythropoietic failure, marrow cellularity, p53 protein accumulation, *WT1* gene expression, and serum levels of CXCL9 and CXCL10, but not to other covariates including the IPSS-R score. The presence of bone marrow fibrosis grade 2 or higher was associated with the presence of mutations in cohesin complex genes (31.5% *vs*. 5.4%, *p*=0.006). By contrast, mutations in *CALR*, *JAK2*, *PDGFRA*, *PDGFRB*, and *TP53* were very rare. Survival analysis showed that marrow fibrosis grade 2 or higher was a relevant significant predictor for of overall survival, and independent of age, performance status, and IPSS-R score in multivariate analysis.

## INTRODUCTION

Bone marrow fibrosis (MF) may arise from a variety of causes [1] and a European consensus has been reached to minimize inter-observer variability [2]. The best known cause of bone marrow fibrosis is the myeloid proliferative neoplasm (MPN) known as primary myelofibrosis (PMF) [3]. However, MF is also found in 10-20% of patients with myelodysplastic syndromes (MDS) [4–9]. MDS with bone marrow fibrosis is not recognized as a distinct entity in WHO 2008 classification [3] and its prognostic role in MDS remains unresolved [6–12].

Both MPN and MDS usually display an hypercellular bone marrow, but one or more peripheral blood cytopenias are commonly found in MDS. Constitutional activation of Jak-Stat pathway, secondary to activating mutations in *JAK2*, *CALR* or *MPL* genes, is a prominent feature of MPN, including PMF [13], but these mutations are infrequent in MDS [14–18]. Neither polycythemia vera nor primary thrombocytemia show MF at diagnosis despite the frequent presence of *JAK2* mutations, and inhibition of the Jak-Stat pathway by ruxolintinib does not reduce MF in PMF, what argues against a pathogenetic role of this pathway in MF. Therefore, the pathogenetic events that underlie MF in MDS and PMF appear to be unrelated to Jak-Stat and yet undisclosed. A multidimensional analysis of MDS taking European Consensus MF grading and mutational analysis into consideration, has not been performed so far.

The aim of this study was to evaluate the biological and clinical events that underlie bone marrow fibrotic changes in MDS as well as to assess its independent prognostic role in a prospective cohort of primary MDS patients.

## RESULTS

### Clinico-biological correlates of bone marrow fibrosis

Bone marrow fibrosis grade 2 or higher was observed in 17 (22.1%) patients. Non-parametric tests showed bone marrow fibrosis grade (MF-0 to MF-3 following European consensus) to be significantly positively correlated (Table 1) with the number of red cell concentrates transfused in the first 16 weeks from diagnosis, ferritin levels, and erythropoietin (EPO) levels, marrow cellularity, abnormal localization of immature precursors (ALIP), p53 protein immunohistochemical (IHC) score, and peripheral blood Wilms’ tumor gene (*WT1*) expression. Conversely there were significant negative nonparametric correlations with the proportion of erythroid precursors in bone marrow aspirates, hemoglobin levels and serum levels of CXCL9 (C-X-C motif ligand 9, also known as monokine induced gamma interferon, MIG) and CXCL10 (C-X-C motif ligand 10, also known as interferon gamma-induced protein, IP10), but not with the other covariates, including the proportion of marrow blast cells or ringed sideroblasts, IPSS-R score, soluble p53 protein (p53), TNF alpha, and interleukins (IL) 6, 1 beta and 8 (C-X-C motif ligand 8, CXCL8) or Schanz's cytogenetic score [19] (more specifically, none of our MF-2/3 patients had either a score of either Very poor or Poor; it was Good in 12 patients and Intermediate in 5). *WT1* gene expression had a significant nonparametric correlation (+0.479) with IPSS-R score.

**Table 1:** Nonparametric correlation between MF grade and other covariates PRBC, packed red blood cells concentrates; wk, weeks.

	N	Spearman's Rho	*p*-value
Age at diagnosis	77	−0.118	0.306
No. of PRBC transfused in first 16 wk	77	+0.306	0.007
**PERIPHERAL BLOOD**			
WBC count (x 10^9^/L)	77	+0.077	0.508
Granulocyte count (x 10^9^/L)	77	−0.008	0.962
Hemoglobin (g/L)	77	−0.283	0.013
Platelet count (x 10^9^/L)	77	−0.005	0.962
Blast cells (%)	77	+0.177	0.124
WT1/GUS ratio (x10^−3^)	77	+0.260	0.023
Ferritin (ng/mL)	77	+0.296	0.009
LDH (U/L)	77	+0.154	0.182
EPO (U/L)	70	+0.341	0.004
Beta2m (mg/L)	77	+0.026	0.822
p53s (U/mL)	76	+0.041	0.723
TNF alpha (pg/mL)	77	−0.064	0.580
IL6 (pg/mL)	77	−0.165	0.153
IL1 beta (pg/mL)	77	−0.038	0.742
CXCL8 (pg/mL)	77	+0.212	0.174
CXCL9 (pg/mL)	75	−0.261	0.024
**BONE MARROW**			
Blast cells at diagnosis (%)	77	+0.061	0.598
Red cell progenitors (%)	77	−0.416	<0.001
Ringed sideroblasts (%)	76	+0.040	0.730
IPSS-R cytogenetic score	77	−0.045	0.695
Cellularity (%)	77	+0.418	<0.001
p53 score	77	+0.230	0.045
**IPSS-R SCORE**	77	+0.062	0.594
**BEJAR's GENE SCORE**	70	+0.235	0.050

### Targeted sequencing

After excluding sequencing errors and all known or possible single-nucleotide polymorphisms, a total of 202 variants were detected in all patients of which 164 were unique (full details are available online as Supplementary Information). These variants including 138 single nucleotide variants (SNV) and 26 insertions/deletions (15 insertions and 11 deletions) were called in 61 genes as somatic changes.

In total, 63 of the 70 patients (90%) whose DNA was of adequate quality harbored at least one mutation (Table 2) with a median of 3 (0-7) mutations per sample including 120 missense variants, 17 stop gained, 14 frameshift variants feature elongation, 9 frameshift variants feature truncation, 2 inframe deletions, 1 inframe insertion and 1 splice donor variant. The most common mutations were p.Lys700Glu in *SF3B1* and p.Pro95His in *SRSF2* observed in 9 patients each, one of them showing both mutations. The *SF3B1* gene was the most frequently mutated in the cohort (27%) followed by *TET2* (25.7%), *RUNX1* (17.1%), *SRSF2*, and *ASXL1* (15.7%). Less common mutations involved *DNMT3A, ZRSR2* (10%)*, CSF3R, CSNK1A1, NOTCH1, KMT2A, SETBP1, STAG2, KRAS,* and *CREBBP,* (Figure S1). *CALR* and *JAK2* mutations were rare in our series (just 1 patient each) and also mutations on *PDGFRA* or *PDGFRB* were infrequent (3 and 1 patients, respectively).

**Table 2:** Mutations observed in 70 MDS patients

Gene	No. of patients with var. (%)	Total no. of variants (*n*=202)	Gene	No. of patients with var. (%)	Total no. of variants (*n*=202)	Gene	No. of patients with var. (%)	Total no. of variants (*n*=202)
*ABL1*	2 (2.9)	2	*GCAT*	3 (4.3)	3	*RAD21*	1 (1.4)	1
*ASXL1*	11 (15.7)	12	*IDH1*	1 (1.4)	1	*RARA*	1 (1.4)	1
*BCOR*	2 (2.9)	2	*IDH2*	2 (2.9)	2	*RET*	2 (2.9)	2
*BCORL1*	2 (2.9)	2	*IRF1*	1 (1.4)	1	*RUNX1*	12 (17.1)	14
*CALR*	1 (1.4)	1	*JAK1*	1 (1.4)	1	*SETBP1*	4 (5.7)	4
*CBLC*	1 (1.4)	1	*JAK2*	1 (1.4)	1	*SF3A1*	1 (1.4)	1
*CDH23*	1 (1.4)	1	*KIT*	1 (1.4)	1	*SF3B1*	19 (27.1)	20
*CDH3*	2 (2.9)	2	*KMT2A*	4 (5.7)	5	*SMC1A*	2 (2.9)	2
*CREBBP*	4 (5.7)	4	*KRAS*	4 (5.7)	4	*SMC3*	1 (1.4)	1
*CSF3R*	4 (5.7)	4	*MECOM*	1 (1.4)	1	*SRSF2*	11 (15.7)	11
*CSNK1A1*	4 (5.7)	4	*NF1*	1 (1.4)	1	*STAG2*	4 (5.7)	4
*CTCF*	1 (1.4)	1	*NOTCH1*	4 (5.7)	4	*SUZ12*	1 (1.4)	1
*CTNNA1*	1 (1.4)	1	*NRAS*	1 (1.4)	2	*TET2*	19 (27.1)	25
*CUX1*	1 (1.4)	1	*NTRK1*	2 (2.9)	2	*TIMM50*	1 (1.4)	1
*DNMT3A*	7 (10)	7	*NUP98*	2 (2.9)	2	*TNFAIP3*	2 (2.9)	2
*ENG*	2 (2.9)	2	*PBRM1*	1 (1.4)	1	*TP53*	3 (4.3)	4
*EP300*	2 (2.9)	2	*PDGFRA*	3 (4.3)	3	*U2AF1*	3 (4.3)	3
*ETV6*	2 (2.9)	2	*PDGFRB*	1 (1.4)	1	*UMODL1*	3 (4.3)	3
*EZH2*	3 (4.3)	3	*PHF6*	1 (1.4)	1	*ZRSR2*	7 (10)	7
*GATA1*	1 (1.4)	1	*PHLPP1*	1 (1.4)	1			
*GATA2*	1 (1.4)	1	*PTPN11*	1 (1.4)	1			

### Associations of mutations and pathways

**Figure 1:**
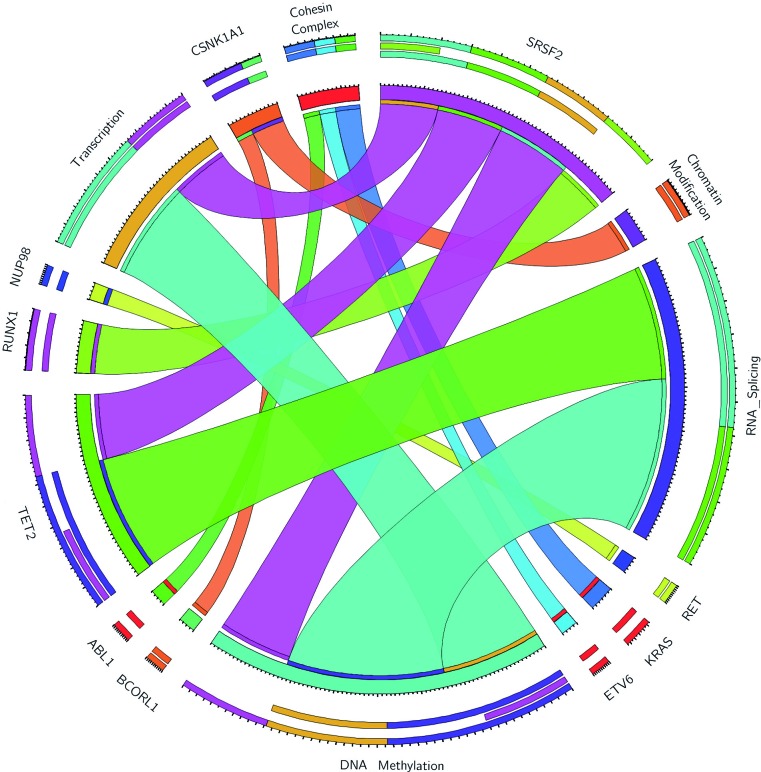
Circos plot of statistically significant associations among mutations in 70 MDS patients, grouped by functional pathways

Mutated genes were grouped into several functional pathways, which have been linked to MDS pathogenesis (Table S1). Amongst these, the most frequent target was RNA splicing, with mutations observed in 51.4% of cases, followed by signaling pathways (48.9%), DNA methylation (38.6%), chromatin modification (37%), transcription (31.4%), and cohesin complex (12.9%). Cohesin complex genes (*CTCF, RAD21, SMC1A, SMC3* and *STAG2*) mutations were always mutually exclusive, and associated with mutations in *KRAS* (*p* = 0.006), *ABL1* (*p* = 0.015) and *ETV6* (*p* = 0.015) (Figure 1).

### Clinico-biological correlates

Patients in the Poor/very poor IPSS-R score category had a higher number of mutations in genes involved in transcription regulation (*p* = 0.026, for trend), and those with a lower Schanz's cytogenetic score [19] (Very Good/ good *vs*. Intermediate *vs*. Poor/very poor) had a higher number of mutations in chromatin modification pathway (*p* = 0.036, for trend). Interestingly, patients with MF grade 2 or higher showed a much higher prevalence of mutations in cohesin complex (35.7% *vs*. 5.4%; *p* = 0.006; Odds Ratio 9.8, CI95% 2.0-48.4). However, no associations between cohesin complex mutations and IPSS-R score, underlying cytogenetic abnormalities, WHO classification or other covariates were observed except for a lower proportion of red cell progenitors (24% *vs*. 33%, *p* = 0.043) and a higher number of packed red blood cells transfused in the first 16 weeks from diagnosis (*p* = 0.043) in mutated patients. Bone marrow fibrosis grade 2 or higher was observed in 17 (22.1%) patients. A comparison of the clinico-biological features of MDS with and without relevant bone marrow fibrosis (grade 2 or higher) is shown in as Table S2, panels A and B.

Bejar's mutation score [14] takes into account mutations in 5 genes, namely *TP53, EZH2, ETV6, RUNX1 and ASXL1,* and patients with bone marrow fibrosis grade 2 or higher had higher Bejar's scores (*p* = 0.006, Mann-Whitney), but not higher IPSS-R scores. By contrast, a statistically significant correlation between Bejar's score and IPSS-R score was observed (Rho = 0.323; *p* = 0.006).

All three *TP53mut* cases showed a p53 protein immunohistochemical (IHC) score higher than 5 (median score 164) as well as more than 1% strongly reactive cells (median proportion 55%), whereas these features were present in only 15.2% and 9.1%, respectively, of the *TP53wt* cases.

**Figure 2:**
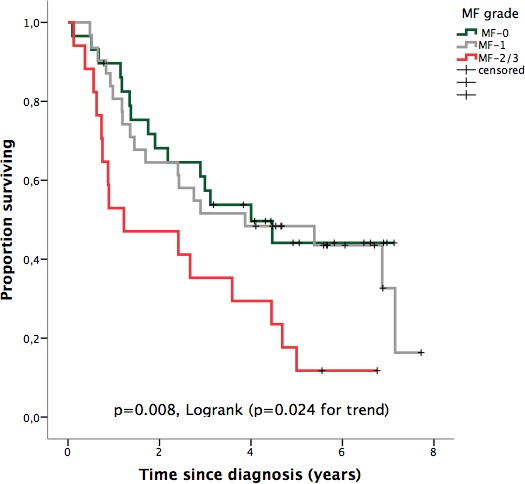
OS according to bone marrow fibrosis grade (European Consensus)

Survival analysis showed that MF grade 2 or higher was a relevant predictor for overall survival (OS) (Figure 2) and independent of age, performance status (PS) and IPSS-R score in multivariate analysis (Table 3). By contrast, the presence of cohesin complex mutations and Bejar's score did not show independent prognostic value for survival, when they were added to the previous model. Censoring for disease-modifying therapies did not change this fact (data not shown).

**Table 3:** Impact of MF on overall survival in MDS patients

		Overall Survival	
Covariate	Univariate	Multivariate	Multivariate
	*p*-value	*p*-value	HR (CI 95%)
**IPSS-R**			
(Very poor/Poor/Intermediate vs. other)	<0.001	<0.001	3.50 (1.76-6.96)
**AGE**			
(Years)	0.047	0.035	1.04 (1.01-1.07)
**PS (ECOG)**			
(0-4)	0.013	0.006	1.61 (1.15-2.25)
**MYELOFIBROSIS GRADE**			
(MF-0/1 vs. MF-2/3)	<0.001	0.002	3.21 (1.53-6.74)
**Bejar's Gene Score for MDS**			
(High risk vs. other)	0.019	0.079	1.75 (0.94-3.25)
**COHESIN COMPLEX MUTATIONS**			
(Any variation)	<0.001	0.115	2.09 (0.84-5.20)

## DISCUSSION

Bone marrow biopsy is a useful tool for the evaluation of MF in MDS, MDS/MPN [20, 21] and other overlapping MF syndromes [22]. Bone marrow reticulin staining predominates in the early stages of MF and it is mainly due to the deposition of hyaluronane [23]. The pathogenesis of this deposition is largely unknown.

MF has been proposed as a poor prognostic factor in both primary and therapy-related MDS [6–10, 12] and it has also been linked to delayed engraftment and worse survival after bone marrow transplantation [24]. However, it has been ruled out as an additive factor for predicting overall survival in MDS by the thorough retrospective analysis that gave rise to IPSS-R, probably, as the authors stated, as a consequence of “the low number of patients assessed for this feature (19%) as well as the variable ways the degree of fibrosis was reported from the different institutions in our study” [11]. Recently, the potential independent impact of MF in MDS has been put again into focus [12] and its genomic correlates have been described [25]. However, a multidimensional analysis including MF grade and mutational analysis has not been performed so far.

Our data show that MF grade is correlated with several cyto-histological and clinical parameters of bone marrow dysfunction in MDS patients as well as their overall survival. These multiple correlations suggest that fibrosis is not an epiphenomenon, but a condition linked to pathogenetic events taking place in MDS.

As communicated previously [26], fibrosis was more prominent when cellularity was very high and also when ALIP was present. Nevertheless, these covariates did not provide further independent prognostic information for OS when MF/2-3 was included in the Cox model (data not shown).

The presence of *JAK2* mutations is common in MPN [27, 28] and is also frequent in cases with refractory anemia with ring sideroblasts associated with marked thrombocytosis (RARS-T) [29]. Nevertheless, it is not restricted to RARS-T cases since *JAK2-*mutated cases have been described in up to 5% of otherwise typical MDS cases [14, 15, 30]. *CALR* mutations are also common in PMF [17, 18] and have been described in up to 8% of otherwise typical MDS cases [18]. Finally, MPL mutations are found in 5% of PMF cases [18] and about the same proportion of typical MDS cases [15]. In our cohort, *JAK2* and *CALR* mutations were rare (one case each), and we did not find any mutations in the *MPL* gene. No case of RARS-T was included in our series.

*WT1* gene expression has been claimed to be a poor prognostic feature in MDS [31], but in our study, it did not provide independent prognostic information (data not shown), probably as a consequence of its correlation with the IPSS-R score. None of our patients had mutations of the *WT1* gene.

The Cohesin complex is a proteic ring structure that has several cell functions including sister chromatid cohesion, DNA damage repair and *RUNX1* transcription regulation [32]. Cohesin dysfunction underlies the so-called “cohesinopathies” such as Down's syndrome, and cohesin mutations are not rare in myeloid diseases. They have been described in up to 15% of MDS patients and some of them (*STAG2* and *SMC1A*) may have a prognostic impact on overall survival [15]. In this paper, we report for the first time a strikingly high prevalence of cohesin complex mutations in patients with MDS with fibrosis as compared with those patients without it. This finding requires confirmation in other patient series, but the prospective design is a particular strength of our study. It would have been very interesting to follow the MF grade of the 3 patients in our series that harbored cohesin mutations at diagnosis but did not show prominent MF at that time, but unfortunately that information was not available.

With respect to p53 protein tissue accumulation and *TP53* gene mutations, both are frequent at diagnosis or during disease evolution in MDS patients with isolated deletion of 5q, as well as in those patients harboring high-risk cytogenetics and are associated with poor prognosis [14, 15, 33–44]. Elevated plasma levels of p53 soluble protein have been described in chronic lymphocytic leukemia, in which it may confer poor prognosis [45], but our data do not support such a role in MDS. We have observed that the p53 IHC score is positively correlated with the grade of MF, even despite the tiny proportion of *TP53* mutations in our patients. This fact suggests that alternative mechanisms for p53 protein accumulation function in a small proportion of MDS cases (e.g. MDM2 protein degradation due to RPS14 haploinsuficiency in 5q deletion patients), which may be eventually detected by checking for the p53 IHC score. In contrast to recently reported findings [25] we did not observe a high prevalence of *TP53* mutations in our MDS patients with fibrosis despite using FISH in cases with fewer than 20 metaphases available (as stated in GESMD guidelines): in fact, only 3 patients out of the 71 patients of the cohort had a *TP53* mutation, of which two were MF-1 and one MF-2. Since none of our MF-2/3 patients had a Poor/very poor IPSS-R cytogenetic risk score, Loghavi's [25] findings might be explained by the higher prevalence of Poor/very poor risk IPSS-R cytogenetic categories (24.2%) that was present in their series.

We have observed a negative correlation between bone marrow fibrosis grade and serum levels of the IFN-γ induced cytokines CXCL9 (an antifibrotic monokine secreted by macrophages) and CXCL10 (another monokine secreted by several cell types including by macrophages, endothelial cells and fibroblasts). Breakdown fragments of hyaluronan generated during tissue injury synergise with gamma-interferon for macrophage expression of CXCL9 *via* NF-kB [46]. Interestingly, both CXCL9 and CXCL10 but not CXCL8, exert their effects *via* chemokine receptor CXCR3, and CXCR3-deficient mice are known to develop progressive interstitial pulmonary fibrosis [47]. These findings suggest that CXCL9 and CXCL10 might be involved pathogenetically in the fibrosis of MDS, although their precise role needs to be better established by functional analyses.

In our study, the Bejar's gene score correlated with both IPSS-R score and bone marrow fibrosis grade. This fact may account for its lack of independent prognostic value in our prospective cohort, in which both IPSS-R and bone marrow fibrosis were considered in the model. Cohesin complex mutations are neither correlated with IPSS-R score nor are they included in the Bejar's score, but they did not have an independent prognostic role in our study.

In conclusion, bone marrow fibrosis changes observed in MDS patients are correlated with erythroid failure and p53 protein accumulation, but they are not linked to mutations in *CALR/JAK2*, cytogenetic score or IPSS-R and might be related to cohesin complex mutations. Bone marrow fibrosis grade 2 or higher, evaluated following the European Consensus, is a poor prognostic feature for OS, independently of IPSS-R, age or performance status, and might be as good a predictor as some current gene profiles.

## MATERIALS AND METHODS

### Patients

The inception cohort comprised 200 primary MDS patients recruited prospectively at diagnosis in eight Spanish public hospitals, between June 2006 and October 2010. Among them, 77 patients had a bone marrow biopsy performed according to attending's physician discretion and GESMD recommendations. Comorbidity was not a limitation for performing a bone marrow biopsy if considered important for a precise diagnosis. Patients provided written informed consent for the use of their samples and clinical data, after approval of the study by the Institutional Review Board of each study site. All the procedures followed were in accordance with the 2008 revision of the Helsinki Declaration of 1975. MDS diagnosis, classification and cytogenetic evaluation were done following the Standard Operating Procedures of the GESMD [48]. Clinical monitoring (diagnosis, general patient's condition, PB sample evaluations, BM evaluations, treatment decisions, leukemic evolution and survival) was always performed at the participating hospitals by dedicated physicians affiliated to the GESMD. Prospective follow-up of the MDS findings observed at diagnosis was not part of the objective of this study and consequently they were not repeated at pre-defined intervals.

The main patient characteristics at diagnosis are shown in Table S3, panels A and B. Patients were followed until June 2014 (median follow-up 3 years, range 0.1-7.7 years) and stratified according to IPSS-R. Thirty patients (39.0%) received disease-modifying therapeutic strategies such as azacitidine (*n* = 20; followed by allogeneic bone marrow transplantation in two patients), intensive chemotherapy (*n* = 10; in 3 cases after azacitidine failure; followed by allogeneic bone marrow transplantation in six patients). Twenty out of the 77 patients (26.0%) progressed into acute myeloid leukemia, 49 (63.6%) died, two (2.6%) were lost to follow-up and 26 (33.8%) remained alive at the time of analysis.

### Evaluation of fibrotic changes and P53 protein accumulation

MF was evaluated independently by two pathologists (F.M.I-G and D.S-V.) following European consensus guidelines [2]. Immunohistochemical accumulation of p53 protein [33, 34] was also evaluated in bone marrow biopsy samples as previously described by our group [35]. In short, the positivity (nuclear stain) for p53 in 500 cells was quantified by observing the bone marrow sections at 1000X magnification under a light microscope, including all visible cells in the count, regardless of whether they were parenchymal or stromal. The reactivity of individual cells was classified as negative (0 points), weakly positive (1 point) or strongly positive (2 points). An overall global score was calculated by adding up the values found for the 500 cells (minimum-maximum score, 0-1000 points). Strong IHC positivity was defined as a strong positive reaction in at least 1% of the observed cells [36]. The positive control was a bone marrow sample from a patient with a large-cell lymphoma that overexpressed p53.

### Clinico-biological covariates

Besides clinical data needed to perform MDS diagnosis, WHO classification and IPSS-R stratification, we collected data on i) the so-called differentiating features of IPSS-R [11], namely age, performance status, levels of serum ferritin and beta2-microglobulin (beta2m), and serum lactate dehydrogenase (LDH) activity at diagnosis, ii) other useful predictors of overall survival in MDS such as transfusion dependence and hemoglobin levels [49, 50], serum erythropoietin (EPO) levels [51] and the expression of peripheral blood *WT1* [52, 53], and iii) sp53 levels [45] and plasma levels of potentially relevant cytokines (TNF alpha, IL1beta, IL6) and chemokines (CXCL8/IL8, CXCL9/MIG and CXCL10IP10), which were also simultaneously determined in samples obtained at diagnosis. Plasma was processed in duplicate for sp53 determination at the coordinating centre using a commercially available biotin-conjugate/streptavidin-HRP ELISA kit (Bender MedSystems, Vienna, Austria) with a sensitivity of 0.33 U /mL. As a rule, healthy donors test negative for sp53. Cytokine/chemokine plasma levels were measured also in duplicate by using cytometric bead arrays (Becton-Dickinson, Franklin Lakes, NJ, USA), acquired on a FACScanto flow cytometer and analyzed with FCAP Array software (Becton-Dickinson, Franklin Lakes, NJ). *WT1* gene expression was evaluated by RT-PCR in peripheral blood and calculated as the *WT1/ GUS* ratio as described elsewhere [52].

### Mutational analysis

The mutational analysis by next-generation sequencing was carried out for the 70 (91%) patients whose DNA was of adequate quality (56 of these presented with MF-0/1, and 14 were MF-2/3) and performed in all cases from the peripheral blood leucocytes to avoid the eventual methodological limitations of a fibrotic bone marrow (“dry tap”). Genomic DNA (gDNA) was extracted and quantified using a Qubit DNA BR assay kit or HS assay kit (Life Technologies, Carlsbad, CA) and tested the adequate quality for the sequencing on MiSeq Illumina (San Diego, CA).

### Library design

Target-capture sequencing on an Illumina platform (Illumina, San Diego, CA, USA) was performed across selected exons of 111 cancer-related genes previously related to MDS or IMF according to a Nextera sequencing design using Illumina DesignStudio (Illumina, San Diego, CA, USA) (Table 4).

**Table 4:** Panel of 111 genes included in the study

ABL-1	CDH23	ETV6	JAK3	NTRK1	SALL4	TCL1B
AEBP2	CDH3	EZH2	JARID2	NUP98	SBDS	TERC
ASXL1	CDK2	FBXW7	JKAMP	PBRM1	SETBP1	TERT
ATRX	CDKN2A	FLT3	KDM6A	PDGFRA	SETBP1	TET2
BCOR	CEBPA	GATA1	KIT	PDGFRB	SF1	TGM2
BCORL1	CREBBP	GATA2	KRAS	PHF19	SF3A1	TIMM50
BCR	CSF3R	GCCAT	LUC7L2	PHF6	SF3B1	TNFAIP3
BMI1	CSNK1A1	GNAS	MECOM	PHLPP1	SFPQ	TP53
BRAF	CTCF	HRAS	MLL	PTEN	SH2B3	TYK2
CALR	CTNNA1	IDH1	MLL2	PTPN1	SMC1A	UA2F1
CBFb	CUX1	IDH2	MPL	PTPN11	SMC3	UMODL1
CBL	DNMT3A	IKZF1	mTOR	RAD21	SPARC	USB1
CBLB	EED	IL3	NF1	RARa	SRSF2	WT
CBLC	EGFR	IRF1	NOTCH1	RET	STAG1	WT1
CD177	ENG	JAK1	NPM1	RPS14	STAG2	ZRSR2
CDH13	EP300	JAK2	NRAS	RUNX1	SUZ12	

### DNA sequencing

The genomic library was prepared using 50 ng of gDNA template and was sequenced following Illumina's standardized protocol (Illumina). Briefly, the amplified libraries were purified using AMPure magnetic beads (Agencourt, Brea, CA), quantified and pooled in equimolar amounts. The pool was loaded at 9 pM on one MiSeq flow cell and then subjected to NGS using a MiSeq sequencer (Illumina). The sequencing was performed sequentially from both ends each for 151 cycles. An additional eight cycles were used to read the index.

A minimum quality score of Q30 (corresponding to a 1:1000 error rate) was required for a minimum of 90% of bases sequenced ensuring high-quality sequencing results and runs failing these metrics were excluded. The mean unique depth of coverage across the capture region was 566 and the number of capture regions was 1661 with a 475805 bases sequencing. Obtained sequences were aligned to the reference genome (GRCh37/hg19) using MiSeq Reporter software (Illumina), which detected discrepancies determining their type, such as deletions, insertions and SNPs. To visualize read alignment and confirm the variant calls, Integrative Genomics Viewer version 2.3.26 (IGV, Broad Institute, MA) was used. For reporting, a sequencing coverage of > 100X (bi-directional true paired-end sequencing), a quality of 100, and a variant frequency of 2% were used as cut-offs. To annotate sequence variant, Illumina Variant Studio 2.2 software was used.

Synonymous variants, noncoding variants, or germline polymorphisms present in database of normal genomes (including dbSNP138, the 1000 genomes project, and our in-house database) at a population frequency (MAF) > = 1% were discarded. The variants that were recurrently observed in our cohort and suspected of being sequencing errors by visual inspection on the IGV browser were removed. Among those, missense SNVs whose variant allele frequencies were 45-55 and all the known hotspot mutations were considered somatic mutations. However the variants described in the Catalogue of Somatic Mutations in Cancer database (COSMIC database) were rescued. In order to elucidate the effects of the different variants with no clear clinical significance, we used the PolyPhen-2 and SIFT web-based platforms and the mutations with deleterious and/or probably damaging were also rescued as somatic mutations (15). The remaining variants were considered candidate somatic mutations. All the mutations were confirmed either by resequencing in an amplicon-based approach (GS Junior Roche 454 for mutations present in <20%) or by Sanger (in mutations observed in more than 20%).

High quality of runs was confirmed by Q30 values > 90% and 151 bidirectional cycles were performed, yielding between seven to ten Gigabases of sequencing data. Overall median coverage was 475 reads (range 120-1083 reads). The mean depth of the targeted sequencing study in 111 genes was 456 reads across the entire cohort (*n* = 70).

All assays were performed blinded to the study end points, by technicians who were not involved in patient management and who were unaware of their evolution.

### Sample size considerations and statistical analysis

The sample size estimate was based on the final maximal number of predictors that would be of practical interest for an eventual prognostic model in daily practice: an international prognostic scoring system, patient's age, a measure of patient's general condition, two peripheral blood findings and a pathological finding not included in current scoring systems. That makes a maximum of predictors to be included in the final multivariate model. At least 10 patients per final predictor was considered appropriate for multivariate analysis.

Continuous and ordinal variables were summarized by their median and interquartile range and Mann-Whitney's test was used to analyze differences in their mean rank. Categorical variables were described by counts and relative frequencies, and Fisher's exact test was used to analyze differences in the distribution of them between patient subsets. Nonparametric correlation between covariates of interest was analyzed and calculated by means of Spearman's Rho. Whenever possible, covariates were dichotomized according to clinically relevant cut-offs (upper limit of normal range for LDH, ferritin > 500 ng/mL, beta2m > 3 mg/L, EPO > 150 U/L). In those cases in which that was not possible (sp53, WT1 gene expression, cytokine levels), cut-off values based on data distribution were chosen. Co-mutations among genes of interest are depicted by means of Circos plots.

Overall survival was defined as the time from diagnosis to death (as a result of all causes), last follow-up or study closing date and was plotted using the Kaplan-Meier product-limit method. The log-rank test was used to check the association between categorical predictors and overall survival. Multivariate analysis of factors influencing overall survival was performed by Cox proportional hazards regression analysis. P-values of less than 0.05 were considered to indicate statistical significance. All p-values presented are two-sided. Statistical analyses were performed using SPSS release 20.0 (SPSS Inc., Chicago, IL).

## SUPPLEMENTARY MATERIAL FIGURE AND TABLES




