# Supplementary material for: Bone marrow fibrosis in myelodysplastic syndromes: a prospective evaluation including mutational analysis
Source: Oncotarget. 2016 Apr 26;7(21):30492–503. doi: 10.18632/oncotarget.9026 (PMC5058695; doi:10.18632/oncotarget.9026)
Supplement: Supplementary file 1 [file oncotarget-07-30492-s001.pdf]

Bone marrow fibrosis in myelodysplastic syndromes: a prospective evaluation including mutational analysis

Supplementary Material

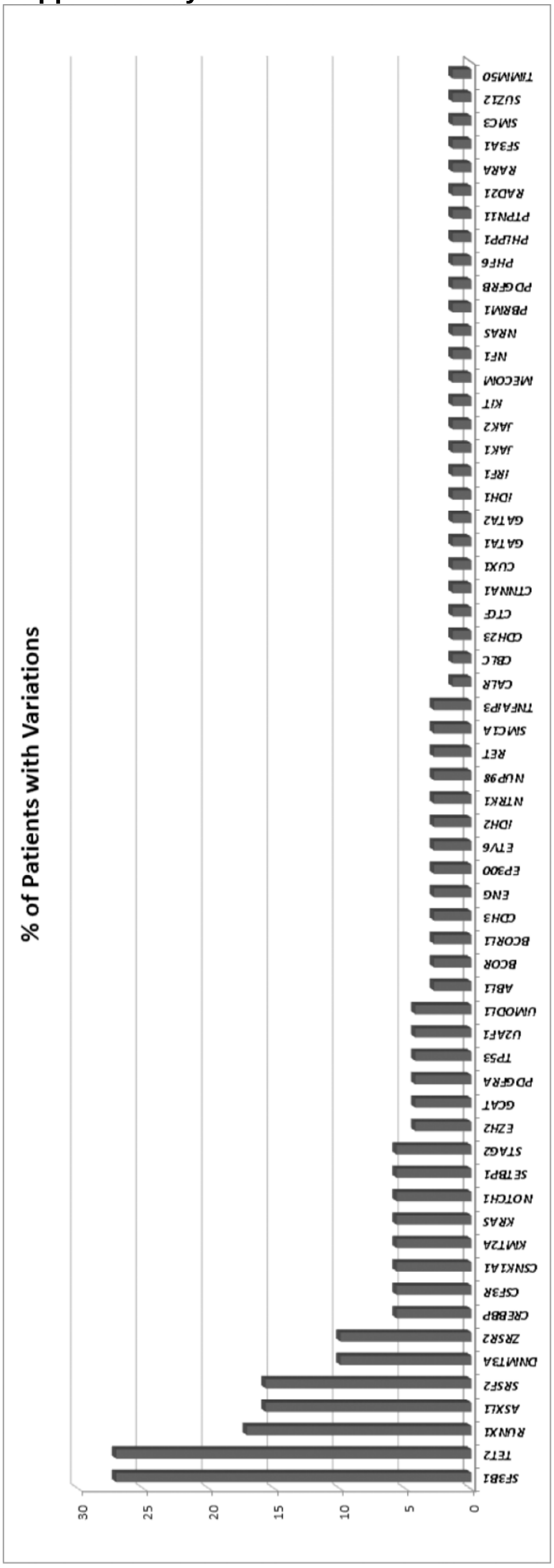

Supplemental Fig. 1. Frequency of gene mutations in 70 MDS patients.

Supplemental Table 1. Genes involved in MDS pathogenesis grouped into functional pathways.

Gene	Functional pathways	Gene	Functional pathways	Gene	Functional pathways
ASXL1	Chromatin modification	***ABL1***	Signalling	***CUX1***	Transcription
BCOR	Chromatin modification	***CBLC***	Signalling	***ETV6***	Transcription
BCORL1	Chromatin modification	***CDH3***	Signalling	***GATA1***	Transcription
CREBPP	Chromatin modification	***CSF3R***	Signalling	***GATA2***	Transcription
EP300	Chromatin modification	***CSNK1A1***	Signalling	***PHF6***	Transcription
EZH2	Chromatin modification	***CTNNA1***	Signalling	***RUNX1***	Transcription
KMT2A	Chromatin modification	***ENG***	Signalling	***TP53***	Transcription
MECOM	Chromatin modification	***IRF1***	Signalling	***UMODL1***	Transcription
PBRM1	Chromatin modification	***JAK1***	Signalling		
SETBP1	Chromatin modification	***JAK2***	Signalling		
SUZ12	Chromatin modification	***KIT***	Signalling		
		KRAS	Signalling		
SF3A1	RNA splicing	***NF1***	Signalling	***DNMT3A***	DNA methylation
SF3B1	RNA splicing	***NOTCH1***	Signalling	***IDH1***	DNA methylation
SRSF2	RNA splicing	***NRAS***	Signalling	***IDH2***	DNA methylation
UA2F1	RNA splicing	***NTRK1***	Signalling	***TET2***	DNA methylation
ZRSR2	RNA splicing	***PDGFRA***	Signalling		
		PDGFRB	Signalling		
CTCF	Cohesin complex	***PHLPP1***	Signalling	***CALR***	Others
RAD21	Cohesin complex	***PTPN11***	Signalling	***CDH23***	Others
SMC1A	Cohesin complex	***RARA***	Signalling	***GCAT***	Others
SMC3	Cohesin complex	***RET***	Signalling	***NUP98***	Others
STAG2	Cohesin complex	***TNFAIP3***	Signalling	***TIMM50***	Others

Supplemental Table 2. Comparative findings between myelodysplastic syndrome cases with or without relevant bone marrow fibrosis. PANEL A. Demographics and peripheral blood findings.

	**MF-0/1**	**MF-2/3**	p-value
No. of patients, (%)	*60* (77.9)	17 (22.1)	
**DEMOGRAPHICS**			
**Age at diagnosis**			
Median,(range), years	77 (70-82)	72 (64.5-80.5)	0.259
**Gender**			
Male, No. of patients, (%)	*38* (63.3)	14 (82.4)	0.240
**Performance status** (ECOG)			
No. of patients, (%)			
0-1	*47 (78.3)*	*12 (70.6)*	*0.840*
2	7 (10.0)	5 (29.4)	
3-4	7 (11.7)	0	
**Transfusion dependence**			
No. of patients, (%)	*15 (25.5)*	*10 (58.8)*	*0.017*
**PERIPHERAL BLOOD**			
**WBC count** (x 10^9/L)			
Median, (IQR)	3.9 (2.5-4.9)	3.5 (2.3-5.2)	0.968
**Haemoglobin** (g/L)			
Median, (IQR)	10.0 (8.9-11.3)	9.2 (7.1-10.3)	0.092
**Platelet count** (x 10^9/L)			
Median, (IQR)	121 (72-246)	156 (81-267)	0.925
**Blast cells** (%)			
Median, (range)	0 (0-0)	0 (0-0.5)	0.133
***WT1/GUS* ratio in PB** (x10^{-4})			
Median, (IQR)	*3* (1.0–13.0)	30 (8.0-85.5)	0.003
**SERUM**			
**Ferritin** (ng/mL)			
Median, (IQR)	309 (173-599)	578 (350-963)	0.025
**LDH** (U/L)			
Median, (IQR)	372 (326-465)	401 (287-514)	0.235
EPO (U/L)			
Median, (IQR)	58 (18-153)	134 (43.3-651.0)	0.067
**Beta2-m** (mg/L)			
Median, (IQR)	*3.0* (2.2-4.5)	3.2 (2.5-3.6)	0.978
**sp53** (U/mL)			
Median, (IQR)	1.6 (0-3.4)	1.7 (1.1-2.9)	0.593
**TNF alpha** (pg/mL)			
Median, (IQR)	0 (0-0)	0 (0-0)	0.504
**IL6** (pg/mL)			
Median, (IQR)	0 (0-6.9)	0 (0-0)	0.152
**IL1 beta** (pg/mL)			
Median, (IQR)	0 (0-0)	0 (0-0)	0.732
**CXCL8** (pg/mL)			
Median, (IQR)	0 (0-20.1)	0 (0-8.7)	0.174
**CXCL9** (pg/mL)			
Median, (IQR)	0 (0-164.2)	0 (0-0)	0.015
**CXCL10** (pg/mL)			
Median, (IQR)	0 (0-137.8)	0 (0-0)	0.055

Supplemental Table 2. Comparative findings between myelodysplastic syndrome cases with or without relevant bone marrow fibrosis. PANEL B. Other parameters of interest. HR=High risk ratio. BMT= Blood and marrow transplantation.

	**MF-0/1**	**MF-2/3**	p-value
No. of patients, (%)	*60* (77.9)	17 (22.1)	
**BONE MARROW ASPIRATION**			
**Blast cells at diagnosis** (%)			
Median, (IQR)	2.0 (0.6-4.8)	1.6 (0.9-4.2)	0.681
**Red cell progenitors** (%)			
Median, (IQR)	35 (23.6-45.2)	24.2 (15.4-32.5)	0.050
**BONE MARROW BIOPSY**			
**Cellularity** (%)			
Median, (IQR)	55 (40-75)	80 (60-92.5)	0.006
**p53 IHC score**			
Median, (IQR))	1 (0-3)	2 (1.5-8.5)	0.027
**Strong p53 reactivity (>1%)**			
No. of patients, (% out of 76)	7 (11.9)	4 (26.7)	0.217
**ALIP**			
No. of patients, (%)	15 (25.0)	11 (64.7)	0.004
**SCHANZ'S CYTOGENETIC STRATA**			
No. of patients, (%)			
Very good/good	43 (71.7)	12 (70.6)	0.458
Intermediate	7 (11.7)	5 (29.4)	
Poor/very poor	10 (16.7)	0	
**IPSS-R CATEGORY**			
No. of patients, (%)			
Very good/good	36 (60.0)	12 (70.6)	0.617
Intermediate	11 (18.3)	2 (11.8)	
Poor/very poor	13 (21.7)	3 (17.6)	
**FUNCTIONAL PATHWAYS**			
No. of patients, (%)	*56 (80.0)*	*14 (20.0)*	
Splicing	*27 (48.2)*	*9 (64.3)*	*0.374*
Signalling	*26 (46.4)*	*8 (57.1)*	*0.557*
Chromatin modification	*19 (33.9)*	*7 (50.0)*	*0.356*
Transcription regulation	*16 (28.6)*	*6 (42.9)*	*0.344*
Methylation	*20 (35.7)*	*7 (50.7)*	*0.368*
Cohesin complex	*3 (5.4)*	*5 (35.7)*	*0.006*
Bejar's HR score	*17 (30.4)*	*9 (64.3)*	*0.029*
**THERAPY**			
No. of patients, (%)			
Azacitidine	15 (25.5)	5 (29.4)	0.758
Intensive chemotherapy	7 (11.7)	3 (17.6)	0.683
BMT	6 (10.0)	2 (11.8)	0.834

Supplemental Table 3. Main patient characteristics at diagnosis. PANEL A. Demographics and peripheral blood findings.

**DEMOGRAPHICS**	
**Age at diagnosis**	
Median,(range), years	76 (31-88)
**Gender**	
Male, No. of patients, (%)	52 (67.5)
**Performance status** (ECOG)	
No. of patients, (%)	
0-1	59 (76.6)
2	11 (14.3)
3-4	7 (9.1)
**Transfusion dependence**	
No. of patients, (%)	27 (35.1)
**PERIPHERAL BLOOD**	
**WBC count** (x 10^9/L)	
Median, (range)	3.9 (0.9-15.4)
IQR	*2.5-4.9*
**Haemoglobin** (g/L)	
Median, (range)	9.9 (5.9-16.1)
IQR	8.6-13.0
**Platelet count** (x 10^9/L)	
Median, (range)	122 (9-1067)
IQR	72-247
**Blast cells** (%)	
Median, (range)	0 (0-5)
IQR	0-0
**WT1/GUS ratio in PB** (x10^{-3})	
Median, (range)	0.5 (0-654.5)
IQR	1.0-31.0
**SERUM**	
**Ferritin** (ng/mL)	
Median, (range)	375 (27-1495)
IQR	189-633
**LDH** (U/L)	
Median, (range)	377 (120-922)
IQR	319-473
EPO (U/L)	
Median, (range)	59 (7-1536)
IQR	21-175
**Beta2-m** (mg/L)	
Median, (range)	3.1 (1.3-12.7)
IQR	2.2-4.0
**sp53** (U/mL)	
Median, (range)	1.70 (0-6.25)
IQR	0-3.05
**TNF alpha (**pg/mL)	
Median, (range)	0 (0-195.6)
IQR	0-0
**IL6 (**pg/mL)	
Median, (range)	0 (0-111.1)
IQR	0-7.6
**IL1 beta** (**(**pg/mL)	
Median, (range)	0 (0-160.3)
IQR	0-0
**CXCL8** (**(**pg/mL)	
Median, (range)	0 (0-339.7)
IQR	0-19.4
**CXCL9 (**pg/mL)	
Median, (range)	0 (0-2204.2)
IQR	0-341.0
**CXCL10** (**(**pg/mL)	
Median, (range)	0 (0-893.3)
IQR	0-125.8

Supplemental Table 3. Main patient characteristics at diagnosis. PANEL B. Bone marrow, classification, IPSS-R and OS.

**BONE MARROW ASPIRATION**	
**Blast cells at diagnosis (%)**	
Median, (range)	2.0 (0-19)
IQR	0.6-4.9
**Red cell progenitors** (%)	
Median, (range)	33 (7.8-64.0)
IQR	23.2-41.5
**SCHANZ'S CYTOGENETIC STRATA**	
No. of patients, (%)	
Very good	4 (5.2)
Good	51 (66.2)
Intermediate	12 (15.6)
Poor	5 (6.5)
Very poor	5 (6.5)
**BONE MARROW BIOPSY**	
**Cellularity** (%)	
Median, (range)	*60 (1-95)*
IQR	*40-80*
**Fibrosis grade** (European consensus)	
No. of patients, (%)	
MF - 0	29 (37.7)
MF - 1	31 (40.3)
MF - 2	15 (19.5)
MF - 3	2 (2.6)
**Fibrosis pattern**	
No. of patients, (%)	
Absent	28 (36.4)
Focal	21 (27.3)
Diffuse	28 (36.4)
**ALIP**	
No. of patients, (%)	26 (33.8%)
**p53 IHC score**	
Median, (range)	1 (0-460)
IQR	1-4
**Strong p53 IHC reactivity (>1%)**	
No. of patients, (% out of 76)	11 (14.5)
**WHO CLASSIFICATION CATEGORY**	
No. of patients, (%)	
RCUD	2 (2.6)
RARS	4 (5.2)
RCMD	40 (51.9)
RAEB-1	15 (19.5)
RAEB-2	7 (9.1)
5q-	7 (9.1)
MDS-U	2 (2.6)
**IPSS-R CATEGORY**	
No. of patients, (%)	
Very good	15 (19.5)
Good	33 (42.9)
Intermediate	13 (16.9)
Poor	10 (13.0)
Very poor	6 (7.8)
**OVERALL SURVIVAL**	
MF-0	
No. of patients, (%)	*29 (37.7)*
Median (years), CI95%	*4.0 (1.92-6.09)*
MF-1	
No. of patients, (%)	*31 (41.3)*
Median (years), CI95%	*3.9 (0.29-7.46)*
MF-2/3	
No. of patients, (%)	*17 (22.1)*
Median (years), CI95%	*1.2 (0-3.29)*

Supplemental Table 4. WHO subtype distribution in cohesin complex mutated and non-mutated cases.

RCUD, Refractory cytopenia with unilineage dysplasia; RARS, Refractory anemia with ring sideroblasts; RCMD, refractory cytopenia with multilineage dysplasia; RAEB-1, Refractory anemia with excess blasts type 1; RAEB-2, Refractory anemia with excess blasts type 2; 5q-, MDS with isolated del(5q); MDS-U, MDS-unclassified.

	**RCUD**	**RARS**	**RCMD**	**RAEB-1**	**RAEB-2**	**5q-**	**MDS-U**	Total
**Cohesin complex non-mutated**	2	3	32	11	6	6	2	62
**Cohesin complex mutated**	0	0	4 (11.1%)	3 (21.4%)	0	1 (14.3%)	0	8
Total	2	3	36	14	6	7	2	70

Supplemental Table 5. IPSS-R strata distribution in cohesin complex mutated and non-mutated cases.

	**Very good**	**Good**	**Intermediate**	**Poor**	**Very poor**	Total
**Cohesin complex non-mutated**	13	28	8	7	6	62
**Cohesin complex mutated**	1 (47.8%)	3 (9.7%)	3 (27.3%)	1 (14.3%)	0	8
Total	14	31	11	8	7	70
