# Supplementary material for: Bone marrow fibrosis in myelodysplastic syndromes: a prospective evaluation including mutational analysis
Source: Oncotarget. 2016 Apr 26;7(21):30492–503. doi: 10.18632/oncotarget.9026 (PMC5058695; doi:10.18632/oncotarget.9026)
Supplement: Supplementary file 2 [file oncotarget-07-30492-s002.pdf]

Bone marrow fibrosis in MDS: Raw mutational data. Ramos et al.

Gene	Chromosome	Coordinate	Type	Variant	Change	Alt Variant Freq	% patients with variation	UPN
ABL1	9	133729537	snv	G>G/A	p.Ala75Thr	41.98	1,4	20
ABL1	9	133748312	snv	G>G/A	p.Asp344Asn	13.01	1,4	26
ASXL1	20	31022562	insertion	A>A/AC	p.Gly685TrpfsTer33	30.71	1,4	54
ASXL1	20	31022592	snv	C>C/T	p.Arg693Ter	31.65	1,4	3
ASXL1	20	31022639	insertion	G>G/GGC	p.Gly710ProfsTer16	29.74	1,4	24
ASXL1	20	31022737	snv	A>A/T	p.Asp741Val	51.41	1,4	51
ASXL1	20	31022898	deletion	TC>TC/T	p.Trp796GlyfsTer22	21.5	1,4	15
ASXL1	20	31022936	deletion	TC>TC/T	p.Pro808LeufsTer10	23.08	1,4	26
ASXL1	20	31022981	deletion	AT>AT/A	p.Leu823Ter	2.27	1,4	36
ASXL1	20	31023717	snv	C>C/T	p.Arg1068Ter	3.83	1,4	13
ASXL1	20	31023821	snv	G>G/T	p.Glu1102Asp	50.57; 43.68	2,8	13; 50
ASXL1	20	31024482	snv	A>A/G	p.Met1323Val	40	1,4	19
ASXL1	20	31024587	snv	C>C/G	p.Pro1358Ala	42.35	1,4	55
BCOR	X	39932235	snv	G>C/C	p.Asn788Lys	99.57	1,4	50
BCOR	X	39934149	insertion	T>T/TC	p.Thr151AsnfsTer35	26.41	1,4	39
BCORL1	X	129150080	snv	C>C/T	p.Thr1111Met	52.39	1,4	12
BCORL1	X	129159070	snv	G>G/A	p.Arg1265His	98.37	1,4	7
CALR	19	13050317	snv	C>C/T	p.Thr90Met	44.33	1,4	15
CBLC	19	45295664	snv	A>A/G	p.Met344Val	47.31	1,4	60
CDH23	10	73406232	snv	G>G/A	p.Ser436Asn	54.98	1,4	2
CDH3	16	68719119	snv	T>T/C	p.Leu479Pro	52.55; 51.41	2,8	23; 25
CREBBP	16	3778068	snv	G>G/A	p.Ala2327Val	39.86	1,4	10
CREBBP	16	3786752	snv	G>G/T	p.His1487Asn	13.36	1,4	53
CREBBP	16	3820723	snv	T>T/C	p.Thr910Ala	43.43	1,4	42
CREBBP	16	3820773	snv	G>G/A	p.Ser893Leu	51.13	1,4	32
CSF3R	1	36932047	snv	C>C/T	p.Glu835Lys	47.82; 38.61	2,9	32; 47
CSF3R	1	36933539	snv	C>C/T	p.Arg583His	38.6	1,4	2
CSF3R	1	36945058	snv	G>G/C	p.Leu14Val	52.43	1,4	29
CSNK1A1	5	148891396	snv	C>C/A	p.Met240Ile	13.46	1,4	8
CSNK1A1	5	148892666	snv	G>G/C	p.Ala216Gly	3.38	1,4	12
CSNK1A1	5	148899890	snv	T>T/G	p.Asp140Ala	6.79; 18.54	2,8	7; 50
CTCF	16	67645067	snv	G>G/A	p.Gly111Glu	51.28	1,4	31
CTNNA1	5	138268312	snv	C>C/T	p.Arg782Cys	48.53	1,4	34

Gene	Chromosome	Coordinate	Type	Variant	Change	Alt Variant Freq	% patients with variation	UPN
CUX1	7	101840555	insertion	G>G/GCCCT	p.Arg635ProfsTer3	11.62	1,4	28
DNMT3A	2	25457243	snv	G>G/A	p.Arg882Cys	31.64	1,4	28
DNMT3A	2	25457249	snv	T>T/C	p.Met880Val	6.83	1,4	17
DNMT3A	2	25463297	deletion	AAAG>AAAG/A	p.Phe732del	16.23	1,4	6
DNMT3A	2	25463584	snv	G>G/C	p.Pro700Ala	9.86	1,4	45
DNMT3A	2	25466797	snv	C>C/T	p.Val636Met	32.38	1,4	15
DNMT3A	2	25469146	snv	C>C/T	p.Asp438Asn	44.61	1,4	41
DNMT3A	2	25469966	insertion	T>T/TA	p.Tyr359LeufsTer34	50.43	1,4	10
ENG	9	130588091	snv	C>C/T	p.Gly191Asp	46.38; 45.88	2,8	40; 63
EP300	22	41527461	snv	C>C/A	p.Pro451His	45.3	1,4	6
EP300	22	41542780	snv	T>T/G	p.Ser697Arg	46.79	1,4	60
ETV6	12	12022522	snv	C>C/T	p.Arg210Cys	50.11	1,4	24
ETV6	12	12022879	snv	G>G/A	p.Ala329Thr	43.49	1,4	26
EZH2	7	148507456	snv	G>G/T	p.Tyr666Ter	81.71	1,4	55
EZH2	7	148515122	insertion	G>G/GCCGT	p.Leu363ThrfsTer5	19.11	1,4	24
EZH2	7	148543610	insertion	C>C/CTG	p.Gln66HisfsTer7	14.09	1,4	7
GATA1	X	48650344	snv	C>C/T	p.Pro105Leu	41.09	1,4	37
GATA2	3	128204960	snv	G>G/C	p.Pro161Ala	48.53	1,4	39
GCAT	22	38206126	snv	G>G/C	p.Ala123Pro	10.97	1,4	48
GCAT	22	38212624	snv	C>C/T	p.Arg413Trp	48.66; 51.15	2,8	14; 30
IDH1	2	209113113	snv	G>G/T	p.Arg132Ser	9.2	1,4	58
IDH2	15	90631934	snv	C>C/T	p.Arg140Gln	46.71; 29.71	2,8	16; 40
IRF1	5	131825088	snv	T>T/A	p.Asn28Ile	9.49	1,4	47
JAK1	1	65313323	snv	G>G/C	p.Ile597Met	49.13	1,4	27
JAK2	9	5073770	snv	G>G/T	p.Val617Phe	8.87	1,4	57
KIT	4	55594279	snv	C>C/A	p.Thr661Asn	4.08	1,4	11
KMT2A	11	118344005	snv	A>A/G	p.Ile711Val	48.78	1,4	38
KMT2A	11	118352565	snv	G>G/T	p.Ser1257Ile	48.61	1,4	1
KMT2A	11	118355592	snv	G>G/A	p.Glu1412Lys	44.89	1,4	42
KMT2A	11	118373307	snv	G>G/A	p.Gly2234Arg	44.62	1,4	38
KMT2A	11	118375572	snv	G>G/A	p.Glu2989Lys	48.74	1,4	39

Gene	Chromosome	Coordinate	Type	Variant	Change	Alt Variant Freq	% patients with variation	UPN
KRAS	12	25398262	snv	C>C/A	p.Leu19Phe	9.42	1,4	55
KRAS	12	25398281	snv	C>C/T	p.Gly13Asp	8.85	1,4	30
KRAS	12	25398282	snv	C>C/A	p.Gly13Cys	8.4	1,4	59
KRAS	12	25398284	snv	C>C/T	p.Gly12Asp	5.08	1,4	31
MECOM	3	168861536	snv	C>C/A	p.Ala154Ser	48.41	1,4	60
NF1	17	29557906	snv	A>A/C	p.Asn1054His	5.34	1,4	59
NOTCH1	9	139390585	snv	C>C/T	p.Val2536Ile	48.89	1,4	48
NOTCH1	9	139390941	snv	G>G/A	p.Pro2417Leu	43.33; 48.58	2,8	13; 27
NOTCH1	9	139399132	snv	C>C/T	p.Val1671Ile	58.2	1,4	5
NRAS	1	115256529	snv	T>T/G	p.Gln61Pro	9.33	1,4	59
NRAS	1	115258747	snv	C>C/A/T	p.Gly12Val/Asp	5.56	1,4	59
NTRK1	1	156830779	snv	G>G/A	p.Gly18Glu	44.72; 50.86	2,9	12; 48
NUP98	11	3726454	snv	G>G/A	p.His1020Tyr	40.43	1,4	21
NUP98	11	3727796	snv	T>T/G	p.Asp935Ala	48.51	1,4	53
PBRM1	3	52582243	snv	G>G/A	p.Arg1529Cys	38.16	1,4	8
PDGFRA	4	55127448	snv	G>G/A	p.Gly79Asp	39.58; 49.32	2,8	9; 24
PDGFRA	4	55151579	snv	G>G/C	p.Gly789Arg	46.87	1,4	27
PDGFRB	5	149499072	snv	C>C/T	p.Arg919Gln	44.65	1,4	63
PHF6	X	133547928	snv	G>G/T	p.Glu221Ter	20.89	1,4	53
PHLPP1	18	60383883	snv	T>T/C	p.Ser323Pro	44.65	1,4	49
PTPN11	12	112910793	snv	G>G/A	p.Gly268Ser	46.23	1,4	38
RAD21	8	117859898	deletion	GAT>GAT/G	p.Ile579GlnfsTer4	7.03	1,4	30
RARA	17	38506138	snv	C>C/T	p.Arg144Ter	50	1,4	25
RET	10	43613833	snv	C>C/T	p.Pro766Leu	59.83	1,4	21
RET	10	43613907	snv	T>T/A	p.Tyr791Asn	48.29	1,4	53

Gene	Chromosome	Coordinate	Type	Variant	Change	Alt Variant Freq	% patients with variation	UPN
RUNX1	21	36206775	snv	G>G/A	p.Thr246Met	41.33	1,4	4
RUNX1	21	36231790	snv	A>A/C	p.Asp198Glu	36.59	1,4	49
RUNX1	21	36231792	snv	C>C/T	p.Asp198Asn	40.82	1,4	38
RUNX1	21	36231822	snv	T>T/C	p.Thr188Ala	29.09	1,4	24
RUNX1	21	36252865	snv	C>C/T	p.Arg166Gln	3.29	1,4	49
RUNX1	21	36252866	snv	G>G/A	p.Arg166Ter	41.6	1,4	39
RUNX1	21	36252882	snv	G>G/C	p.Asp160Glu	12.21	1,4	43
RUNX1	21	36252965	insertion	T>T/TCA	p.Met133Ter	5.64	1,4	57
RUNX1	21	36259166	snv	T>T/A	p.Asn109Tyr	33.17	1,4	38
RUNX1	21	36259192	snv	G>G/A	p.Ser100Phe	16.51	1,4	61
RUNX1	21	36259324	snv	A>A/G	p.Leu56Ser	51.4; 61.82; 47.45; 46.96	5,7	1; 16; 22; 27
SETBP1	18	42281579	snv	G>G/A	p.Glu90Lys	3.57	1,4	37
SETBP1	18	42530501	snv	A>A/G	p.His399Arg	51.71	1,4	7
SETBP1	18	42531907	snv	G>G/A	p.Asp868Asn	7.73	1,4	48
SETBP1	18	42532148	snv	G>G/A	p.Arg948His	46.87	1,4	47
SF3A1	22	30741098	snv	T>T/C	p.Ile159Val	2.87	1,4	17
SF3B1	2	198266593	snv	T>T/A	p.Lys748Met	9.81	1,4	30
SF3B1	2	198266834	snv	T>T/C	p.Lys700Glu	37.21; Sanger; 30; 45; 24.66; 40; Sanger; Sanger; Sanger	12,8	[illegible]6; 27; 32; 34; 35[illegible]
SF3B1	2	198267359	snv	C>C/A	p.Lys666Asn	6.97	1,4	21
SF3B1	2	198267360	snv	T>T/C	p.Lys666Arg	32.82; 39.23; 24.07; 7.06	5,7	15; 33; 47; 57
SF3B1	2	198267361	snv	T>T/C	p.Lys666Glu	21.82	1,4	50
SF3B1	2	198267371	snv	G>G/T	p.His662Gln	3	1,4	27
SF3B1	2	198267483	snv	C>C/T	p.Arg625His	20.36	1,4	5
SF3B1	2	198267484	snv	G>G/A	p.Arg625Cys	26.67	1,4	13
SF3B1	2	198267491	snv	C>C/G	p.Glu622Asp	15.2	1,4	41
SMC1A	X	53436388	snv	C>C/T	p.Arg434Gln	39.26	1,4	20
SMC1A	X	53440030	snv	A>A/C	p.Leu225Arg	14.15	1,4	10
SMC3	10	112361545	snv	T>T/C	p.Leu932Pro	4.56	1,4	26
SRSF2	17	74732935	deletion	CGGCGGCTGTGGT GTGAGTCCGGGG> CGGCGGCTGTGGT GTGAGTCCGGGG/C	p.Pro95_Arg103del	8.97; 12.5	2,8	43; 54

Gene	Chromosome	Coordinate	Type	Variant	Change	Alt Variant Freq	% patients with variation	UPN
STAG2	X	123164903	insertion	T>T/TC	p.His73ProfsTer14	26.15	1,4	61
STAG2	X	123184977	insertion	G>G/GAA	p.Val343LysfsTer2	46.88	1,4	59
STAG2	X	123205051	snv	C>C/G	p.Ser804Ter	13.18	1,4	41
STAG2	X	123220440	snv	C>C/T	p.Arg1033Ter	30.37	1,4	24
SUZ12	17	30302660	snv	C>C/G	p.Leu251Val	15.36	1,4	6
TET2	4	106155868	snv	A>A/G	p.Thr257Ala	43.17	1,4	61
TET2	4	106156432	deletion	CTT>CTT/C	p.Leu446LysfsTer8	21.39	1,4	19
TET2	4	106156551	snv	T>T/A	p.Cys484Ter	32.42	1,4	49
TET2	4	106156729	snv	C>C/T	p.Arg544Ter	51.4	1,4	53
TET2	4	106157181	insertion	T>T/TA	p.Met695AsnfsTer17	19.02	1,4	47
TET2	4	106157554	insertion	T>T/TA	p.Tyr819Ter	50.82	1,4	52
TET2	4	106157698	snv	T>T/C	p.Tyr867His	49.44	1,4	12
TET2	4	106158243	deletion	CA>CA/C	p.Lys1049AsnfsTer6	25.72	1,4	49
TET2	4	106158479	insertion	A>A/AG	p.Tyr1128IlefsTer2	27.02	1,4	56
TET2	4	106158509	snv	G>G/A	splice_donor_variant	16.2	1,4	43
TET2	4	106164068	snv	G>G/A	p.Cys1193Tyr	11.03	1,4	43
TET2	4	106164913	snv	C>C/T	p.Arg1261Cys	5.51	1,4	41
TET2	4	106164916	snv	C>C/T	p.Arg1262Trp	30.19	1,4	34
TET2	4	106180790	snv	G>G/A	p.Cys1273Tyr	33.42	1,4	30
TET2	4	106180817	snv	G>G/T	p.Gly1282Val	15.31	1,4	1
TET2	4	106180857	snv	C>C/G	p.Tyr1295Ter	7.21	1,4	57
TET2	4	106190828	snv	C>C/G	p.Ser1369Ter	41.86	1,4	31
TET2	4	106193748	snv	C>C/T	p.Arg1404Ter	19.76	1,4	41
TET2	4	106193931	snv	C>C/T	p.Arg1465Ter	29.72	1,4	30
TET2	4	106196726	snv	C>C/T	p.Gln1687Ter	44.15	1,4	31
TET2	4	106196819	snv	G>G/T	p.Val1718Leu	49.78	1,4	54
TET2	4	106196834	snv	C>C/T	p.Pro1723Ser	53.57	1,4	12
TET2	4	106197167	deletion	CAGGGTGTGGCTTCT> CAGGGTGTGGCTTCT/C	p.Gln1834ArgfsTer7	3.74	1,4	36
TET2	4	106197308	snv	C>C/T	p.His1881Tyr	24.16; 32.83	2,8	19; 22

Gene	Chromosome	Coordinate	Type	Variant	Change	Alt Variant Freq	% patients with variation	UPN
TIMM50	19	39972581	snv	G>G/C	p.Gly159Ala	47.41	1,4	41
TNFAIP3	6	138199760	snv	C>C/T	p.Pro393Leu	58.6; 49.51	2,8	10; 44
TP53	17	7577094	snv	G>G/A	p.Arg282Trp	19.92	1,4	20
TP53	17	7578190	snv	T>T/C	p.Tyr220Cys	22.77	1,4	2
TP53	17	7578282	deletion	GGCCA>GGCCA/G	p.Leu188ProfsTer58	2.51	1,4	2
TP53	17	7578394	snv	T>T/C	p.His179Arg	70.52	1,4	40
U2AF1	21	44514777	snv	T>T/C	p.Gln157Arg	32.49	1,4	47
U2AF1	21	44524456	snv	G>G/A	p.Ser34Phe	26.98; 17.78	2,8	3; 46
UMODL1	21	43504269	snv	C>C/G	p.Pro132Arg	46.56	1,4	47
UMODL1	21	43529782	snv	C>C/A	p.Pro544Thr	42.31	1,4	45
UMODL1	21	43546473	snv	G>G/A	p.Val1184Met	44.37	1,4	8
ZRSR2	X	15818068	deletion	AAG>AAG/A	p.Arg68AlafsTer5	61.05	1,4	9
ZRSR2	X	15827361	insertion	C>C/CGT	p.Asp161TrpfsTer5	31.36	1,4	18
ZRSR2	X	15827389	snv	C>C/T	p.Arg169Ter	6.76; 21.36	2,8	1; 62
ZRSR2	X	15833945	snv	C>C/T	p.Gln235Ter	24.83	1,4	41
ZRSR2	X	15841230	insertion	C>C/CAGCCGG	p.Gly438_Ser439 insSerArg	36.82; 40.78	2,8	40; 51
